# 

**Published:** 1887-08-13

**Authors:** 


### The Hospital.

An Institution, Family, and Parochial Journal of Hospitals, Asylums, and all Agencies for the Care of the Sick, Criticism and News.

SATURDAY, AUGUST 13TH, 1887.

### NOTICE.

ON and after August 1st, 1887, THE HOSPITAL will be published at 2, Paternoster Square, E.C. Mr. A. P. WATT will, from the date named, assume the business management of the Journal, and all communications relative to the Publishing and Advertising Departments should, after August 1st, be sent to him. All accounts due for advertising in THE HOSPITAL from April 1st, 1887, must be paid to Mr. A. P. WATT, whose receipt will be sufficient discharge.

### NOTICE TO CORRESPONDENTS.

All correspondence must be written on one side of the paper only.
We cannot undertake to return MSS. not used.

**Communications**, whether intended for insertion or for private information, must be authenticated by the names and addresses of the writers, not necessarily for publication.

**Local papers** containing reports or news paragraphs **should be marked.**

It is especially requested that early intelligence of **local events of interest**, or which it may be thought desirable to bring under notice, may be sent direct to the Editor.

Communications respecting editorial matters should be addressed to the Editor, Norfolk House, Norfolk Street, Strand, London, W.C.

### THE LITERARY PRIZE.

A PRIZE of **Two Guineas** will be given every month for the best story or incident based upon hospital, or asylum, or student life, or any incident connected with the professions of medicine or nursing.

*** THE CHILDREN'S CORNER and AMUSEMENTS WITH PROFIT will be found on page vi of this week's issue.

### THE CHILDREN'S CORNER.

**Fourth Quarterly Prize Competition.**
Began 2nd July, 1887 ; ends 24th Sept., 1887.

**Puzzles—Seventh Week.**

No. 26. DECAPITATIONS. 1. Behead a certain country and you will find another. 2. What bird beheaded becomes another ? 3. What country beheaded will show you what no one likes ? 4. What stone beheaded becomes a soft substance ?

No. 27. CHARADE.

My first two letters a man,
My first three letters a woman,
My first four a great man,
And my whole a great woman.

No. 28. FRENCH PUZZLE. Convert the following into a French sentence :

P Ja
A

No. 29. ENIGMA.

On Alpine heights they did not climb, but homely English ground ;
Excelsior not their device, but sad the fate they found.
They did not climb for love of fame, but went at Duty's call,
They were united in their aim, divided in their fall.

**Letters—Seventh Week.**

Next week send me an account of the place where you are spending your holidays.

**Results of Fifth Week—Puzzles.**

No. 17. GEOGRAPHICAL ACROSTIC.

| | | |
|---|---|---|
| L | inne | T |
| O | | H |
| N | ew Guine | A |
| D | a | M |
| O | rang | E |
| N | ante | S |

No. 18. *Postman.*

No. 19. If there are four corners in a room, each containing a cat, which is sitting on its own tail, and opposite another cat, there must be *four* cats in the room.

No. 20. *Listen*, transposed, becomes *silent.*

*All right*—Alsie, Scrooge, A. C. K. ; *Three right*—Toby, A. G. S. McCulloch, Umslopogaas, Joe, F. E. R. N., Agatha, Skye, Princess Ida, Ossian, The Eda ; *Two right*—Socrates, Ellie, Mount Edgcumbe ; *One right*—Poodle, Le Cerf Agile, Mikado.

**Letters.**

I have not had my usual number of letters this week. I suppose, as the holidays have just begun, everyone feels a little lazy, and having put lesson-books away for a time, are not inclined to hunt them out again in order to send me a list of *their favourite studies.* Toby (aged 14) writes a most interesting account of his French classes, and I think all who read it will wish they could learn foreign languages in as delightful a manner :—

I think that my favourite study is French, although I am very fond of Mathematics, and have got two prizes for it. In our French class we have a very jolly way of learning the language. We have arranged a Debating Society, and frequently have debates. On the day before the debate, we all write a subject on a slip of paper, and then hand them over to the president, who reads them out in French, and we vote for those that we think most suitable. This goes on till the number of subjects is thinned down to two, when you are not allowed to vote for more than one. The voting then takes place, and generally settles the matter, but sometimes there are exactly the same number for both, and then the subject is chosen by lot. On the following day we divide ourselves into "government" and "opposition," viz., those who intend to support the motion, and those who intend to oppose it. The President, who is the master of the form, sits at a raised desk on a platform. He then calls on the opener, who puts forward the motion, and supports it with a short speech in French. This is then answered by one of the opposition, and so on till all have spoken. A division then takes place, and if the government gets most votes the motion is passed ; if otherwise, it fails. This makes the lesson very interesting, and I always look forward to the French afternoon.

Mikado is very musical, and belongs to a Choral Society. Le Cerf Agile is devoted to flowers. Skye prefers Latin, Drawing, and Geography ; whilst Socrates goes in for Painting, Drawing, Chemistry, Shakespeare, and Euclid.

Three marks each :—Toby, Skye, Mount Edgcumbe, Ossian, Princess Ida. Two marks each :—Mikado, Socrates, Le Cerf Agile.

N.B.—Pressure of space prevents the insertion of more letters.

Answers must be addressed to the "PUZZLE EDITOR," THE HOSPITAL, Norfolk House, Norfolk Street, Strand, W.C., not later than Thursday next. The "Children's Corner" Coupon (see advertisement pages) must be enclosed.

### AMUSEMENTS WITH PROFIT.

### PRIZE COMPETITIONS.

**Quarterly Prize Competition.**
Began 23rd July ; ends 15th October.

**No. 7. Double Acrostic.**

I.

My first, great master of the solemn strain,
Sway'd with a prophet's rod the choral train ;
And, while he proved his own expressive art,
Upraised to heav'n a nation's ravished heart.

II.

He, waif of revolutions, Poland's child,
But touch'd the keys, in strange, sweet notes and weird,
And died, swept down by human tempests wild,
Ere yet the echo of his strains he heard.

1. An Arian Vandal at whose fierce commands
Confessors sixty lost their tongues and hands

2. And are crusaders free,
To break faith with the Turk ?
This Kalif taught them honesty,
By Varna's bloody work.

3. This little word,
When from a loved one's lips,
Love hopes its sanction may be heard,
Is hope's eclipse.

4. The Athanasius of this age,
Great Orleans' prelate, stemmed the rage
Of tyranny's advance,
And won the cause of liberty,
Holding the truth that makes men free
For God, the Church, and France.

5. Nothing to do ; and doing it badly ;
Life not worth living ; and living it sadly ;
Wearied and listless ; utterly bored ;
What is the matter ? That is my word.

6. ————"The miserable sire,
Wrapt with his sons in Fate's severest grasp,
The serpents, twisting round, their stringent folds
Inextricable tie."

**Enigma.**

I stand alone on the stormy heath,
(The raven above and the serpent beneath),
Who passes me by, tho' bright be the day,
But glanceth a moment, then hastens away.
And oft in the shade of the moon's sickly beams
I've filled the beholder with terrible dreams ;
Though outstretched my hand no traveller takes it,
'Tis friendly to all, yet nobody shakes it,
But while timid man hurries fast out of sight,
The robin will oft on my finger alight.

**Answers.**

No. 3. DOUBLE ACROSTIC.

| | | |
|---|---|---|
| V | alhall | A |
| O | a | R |
| L | o | T |
| U | r | I |
| N | ata | L |
| T | yrre | L |
| E | y | E |
| E | a | R |
| R | ealit | Y |

Correct answers have been received from Man of the Sea, V. W., Perseverance, Gretta, Mater, Condorcet, Brownie.

No. 4. THE POWER OF ASSOCIATION.

The short essays received show considerable ability in dealing with the subject in its varied aspects. Everyone is unanimous in wishing well to, and acknowledging the power for good of The Hospitals Association.

Answers have been received from Brownie, Gretta, Mater, Ellice, V. W., Condorcet, Aralc, Perseverance.

**The Prize Winners.**

As Gretta and Dawnlover have agreed to divide the Prize, we award

GRETTA (Miss Margaretta Parker, Effingham Villa, Park Lane, Stoke Newington, N.), 1½ guinea.

DAWNLOVER (Rev. J. E. L. Nowers, 4, Elsworthy Road, London, N.W.), 1½ guinea.

Answers must be addressed to THE PRIZE EDITOR, AMUSEMENTS WITH PROFIT, THE HOSPITAL, Norfolk House, Norfolk Street, Strand, W.C., not later than the *first* post on Friday next. The full name and address must in all cases be given, but a *nom de plume* may be added for publication. Only one side of the paper should be written on. The "Amusements with Profit" Coupon (see advertisement pages) must be enclosed with replies.

